# Hyperparameter Tuning and Pipeline Optimization via Grid Search Method and Tree-Based AutoML in Breast Cancer Prediction

**DOI:** 10.3390/jpm11100978

**Published:** 2021-09-29

**Authors:** Siti Fairuz Mat Radzi, Muhammad Khalis Abdul Karim, M Iqbal Saripan, Mohd Amiruddin Abd Rahman, Iza Nurzawani Che Isa, Mohammad Johari Ibahim

**Affiliations:** 1 Department of Physics, Faculty of Science, Universiti Putra Malaysia, Serdang 43400, Malaysia; sitifairuz9609@gmail.com (S.F.M.R.); mohdamir@upm.edu.my (M.A.A.R.); 2 Department of Computer and Communication Systems Engineering, Faculty of Engineering, Universiti Putra Malaysia, Serdang 43400, Malaysia; iqbal@upm.edu.my; 3 Programme of Diagnostic Imaging and Radiotherapy, Universiti Kebangsaan Malaysia, Kuala Lumpur 56000, Malaysia; zawani@ukm.edu.my; 4 Faculty of Medicine, Universiti Teknologi MARA, Sungai Buloh 47200, Malaysia; mji@uitm.edu.my

**Keywords:** machine learning, breast cancer, genetic programming, tree-based pipeline optimization tool

## Abstract

Automated machine learning (AutoML) has been recognized as a powerful tool to build a system that automates the design and optimizes the model selection machine learning (ML) pipelines. In this study, we present a tree-based pipeline optimization tool (TPOT) as a method for determining ML models with significant performance and less complex breast cancer diagnostic pipelines. Some features of pre-processors and ML models are defined as expression trees and optimal gene programming (GP) pipelines, a stochastic search system. Features of radiomics have been presented as a guide for the ML pipeline selection from the breast cancer data set based on TPOT. Breast cancer data were used in a comparative analysis of the TPOT-generated ML pipelines with the selected ML classifiers, optimized by a grid search approach. The principal component analysis (PCA) random forest (RF) classification was proven to be the most reliable pipeline with the lowest complexity. The TPOT model selection technique exceeded the performance of grid search (GS) optimization. The RF classifier showed an outstanding outcome amongst the models in combination with only two pre-processors, with a precision of 0.83. The grid search optimized for support vector machine (SVM) classifiers generated a difference of 12% in comparison, while the other two classifiers, naïve Bayes (NB) and artificial neural network—multilayer perceptron (ANN-MLP), generated a difference of almost 39%. The method’s performance was based on sensitivity, specificity, accuracy, precision, and receiver operating curve (ROC) analysis.

## 1. Introduction

Breast cancer has been recorded as the most frequently diagnosed type of cancer among women. Imaging techniques and assisted cancer diagnosis approaches have been extensively developed to detect and treat breast cancer early to reduce mortality rates [1]. Data mining and computer-aided techniques have been developed for detecting and classifying breast cancer, including several stages: pre-processing, the extraction of functions, and classification [2,3,4]. Pre-processing of mammography, such as improving contrast, is critical in enhancing peripheral region visibility and intensity distribution to enable interpretation and analysis [5]. Feature extraction in the detection of breast cancer is highly important as it helps to differentiate benign from malignant tumors. Upon extraction, the segmentation extracts the image properties such as smoothness, thickness, depth, and regularity [5,6]. While machine learning (ML) has demonstrated several benefits, designing the successful application of a ML framework requires considerable effort from human experts as there is no algorithm that can achieve good performance on all possible problems, as described by the No Free Lunch theorem [7]. Although health researchers are well-acquainted with clinical data, they still often lack in the ML expertise needed to apply these techniques to big data sources. Moreover, the interactive process between data scientist and healthcare researchers requires a large amount of time and effort from both sides. 

As data science becomes increasingly popular, it is necessary for data to be more accessible, flexible, and scalable in order to choose the appropriate and optimized ML model for a given data set. A common approach to obtain an optimized ML is by performing an exhaustive search of the selected algorithm parameter such as the grid search method [8]. Classifiers for ML algorithms typically contain several parameters that need to be selected and optimized [9,10,11]. These parameters are known as hyperparameters and cannot be obtained directly from the data. Similarly, ML model selection comes with different pre-processing algorithms that can be crucial in developing an effective model, such as feature selectors that help to reduce the list of features according to selected statistical score metrics, transforming features that help transform a data set with pre-processing features (such as standardization and standardization), and dimensionality reduction for the set of features or creating new features from existing ones that might be required to enrich signal data. Hence, automated ML or AutoML, a new research area motivated by this mission across industries, has emerged with the goal of automatically optimizing parts of the ML pipeline. 

AutoML assists in eliminating the conjectures from this process by constructing and evaluating considered ML algorithms and pre-processing methods using a search algorithm [12]. In AutoML, there are a range of optimization techniques, among them hyper-parameter ML tuning implemented in mlr R kit; complete pipeline optimization Bayesian hyperparameter used in Auto-WEKA and auto-sklearn; and AutoPrognosis, which offers Bayesian optimization of pipeline operators, including imputer selection (the group of algorithms to substitute missing data for replacement values), selected functional transformers, ML model, and calibrator [13,14,15,16]. ML expertise chooses a suitable method to solve the current problem, but it could be a very challenging task for a non-expert to develop an optimized model that can achieve the desired performance [17]. 

AutoML is the process of automating the end-to-end selection process of ML to real- world problems. The main concern regarding AutoML is a combination where any proposed algorithm is needed to find a suitable combination of operations for each part of the ML pipeline in order to reduce bias. Mathematically, AutoML can be described as follows:

COsOP+2N.G(f1,f2)PNM+∑m′∈M∑r∈RP(⟨m′.r|m⟩P(r+y.v(m′)))

here,

*O_P_* is the default pre-defined operation set;
*O_S_* indicates the operations selected by the algorithms;
*G* (*f*_1_, *f*_2_) represents the generator function for developing new features;
*N* is the number of features selected; and
*N_M_* = maximum number of features to be chosen.

Data pre-processing automation is viewed as a sequence of actions that are selected (*O_S_*) from the default (*O_P_*) operating set and executed in a data set. The features are extracted by choosing the appropriate features (2*N*) from the data set by figuring and generating new (*G* (*f*_1_, *f*_2_)) dependent pairs. The selection of the model and the optimization of the hyperparameters work to find the optimum configuration of the parameter from an infinite search area or learn from previous models designed for specific purposes. The last term of the equation reflects the stochastic learning algorithm that has been used to limit the configuration space for several years [18].

**Figure 1:**
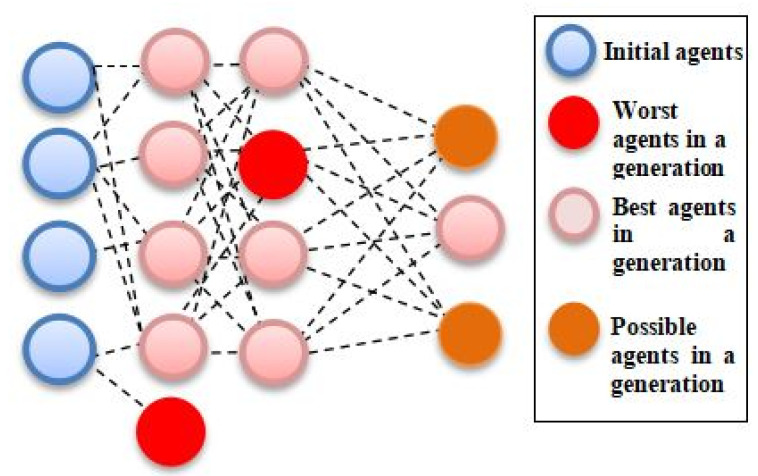
Evolutionary tree-based pipeline optimization tool (TPOT) algorithm, in which each best agent generation generates the next generation.

Here, the tree-based pipeline optimization tool (TPOT) was implemented; it applies an advance approach in the optimization process by adopting genetic programming (GP) to find the optimum ML pipelines. Broadly, TPOT constructs trees of mathematical functions that are optimized with respect to a fitness metric, such as classification accuracy [19]. Each generation of trees is constructed via random mutations to the tree’s structure, or the operations performed at each node in the tree [20]. Repeating this process for a number of training generations produces an optimal tree. It will subsequently develop optimized ML pipelines that can improve as well as surpass the efficiency of other conventional supervised ML algorithms. The pipeline was assessed according to the accuracy of the classifiers at each iteration. Mutation, selection, and crossover operators were used to improve the GP algorithm to find the best pipeline as shown in Figure 1. Hence, the aim of this study was to evaluate the efficacy of TPOT with selected hyperparameter in predictive and its reliability in combined data types and wide feature spaces.

## 2. Materials and Methods

**Figure 2:**
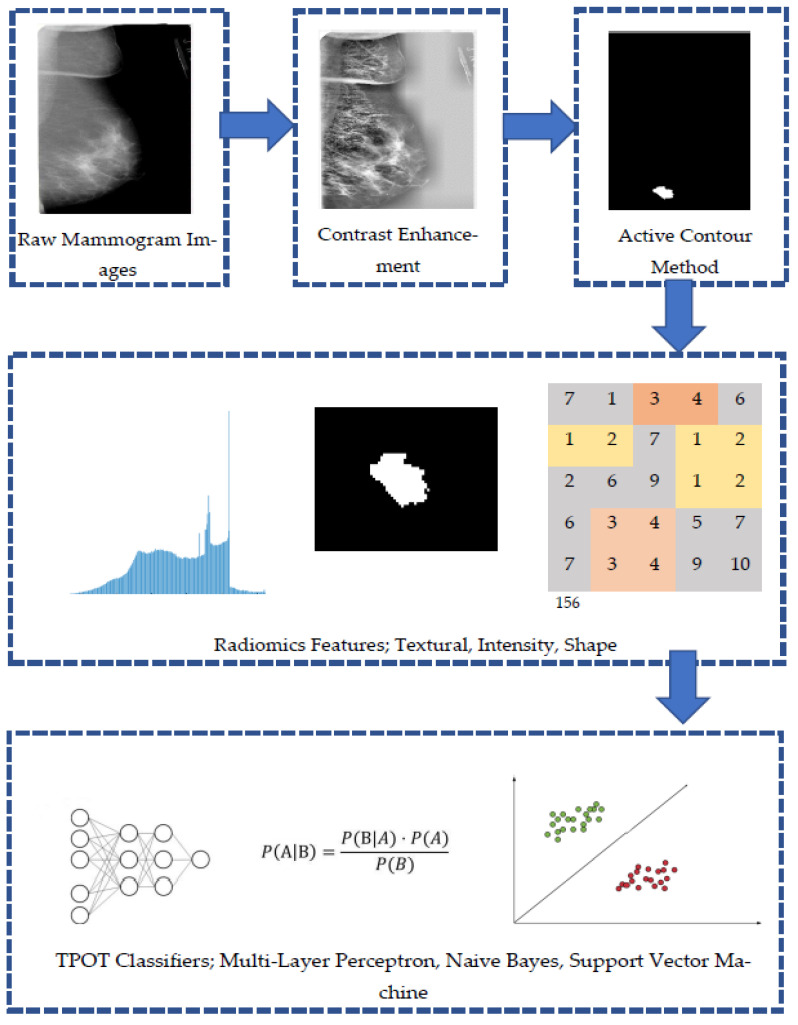
Outline of the proposed method to distinguish between benign and malignant breast mass.

Figure 2 shows an outline of the method proposed in this study. First, the location of the breast tumor was specified on the mammography images, and the lesion was extracted. Next, 29 radiomics features related to information on the shape, texture, and intensity of the lesions were calculated from the extracted images. The accuracy, ROC score, precision, and recall were compared by inputting the obtained radiomics features to various classifiers restricted by TPOT. 

TPOT or tree-based pipeline optimization tools is a computational tool that performs intelligent search over machine learning pipelines that consist of supervised classification models, preprocessors, feature selection techniques, and any other estimator or transformer that follows the scikit-learn API (http://epistasislab.github.io/tpot/, accessed on 5 April 2020). There are several packages that were used to develop TPOT including: NumPy, DEAP, SciPy, scikit-learn, update_checker, tqdm, stopit, pandas, joblib, and xgboost. The package was first installed in Python by using the command: *pip install tpot*, before being imported as an AutoML model. 

The pipeline extracted from TPOT may be composed of various combinations of data transformers provided in the Python library of Scikit-learn, e.g., pre-processors (Min-Max Scaler, Standard Scaler (SS), Max Abs Scaler, Normalizer, Binarizer, and polynomial features expansion) and selectors (Recursive Feature Elimination (RFE), Select Percentile (SP) and Variance Threshold). TPOT also provides several custom features (zero counts, stacking estimator (SE)), a hot encoder, and a range of transformer applications of sklearns. The entire TPOT configuration consisted of 11 classifiers, 14 feature transformers, and 5 feature selectors, all of which combined with TPOT and formed the best pipeline from all of these configurations. TPOT pipeline typically starts with one or more copies of the entire data set at the start of the tree structure and continues with function transformation or feature selectors as illustrated, or with the ML algorithm. Then, the operators adjust the original data set and pass it to the next operator along the tree. In certain cases, a hybrid operator combines the different copies of the data set into a single set.

### 2.1. Image Data Set

The mammogram data set used in this study contained real images from the Curated Breast Imaging Subset of the Digital Database for Screening Mammography (CBIS-DDSM) data set. The CBIS-DDSM data set, available through The Cancer Imaging Archive (TCIA), is an updated and standardized version of the public Digital Database for Screening Mammography (DDSM) data set that was made available in 1997 [21]. It was curated with the help of a trained mammographer who removed images in which the lesion was not clearly seen, or which contained personal information. In this study, 378 images with confirmed diagnoses were presented in a .csv file. A total of 147 cases of benign lesions and 231 cases of malignant lesions were analyzed. The image details are shown in Table 1.

### 2.2. Semiautomatic Segmentation for Region-of-Interest (ROI) 

The mammogram images were enhanced by Contrast Limited Adaptive Histogram Equalization (CLAHE) to improve the quality of the image for better visual and computational analysis before the segmentation process [22,23]. The Active Contour Model (ACM) technique is a semiautomatic iterative region-growing image segmentation algorithm, and the iteration has been set to 200 for every mammogram image. The region of interest in each mammogram image used in this study were confirmed and reviewed by an experienced mammographer.

### 2.3. Extraction of the Radiomic Features

Three types of image features, namely, shape, intensity, and texture, were taken from the segmented tumor ROIs. All image data in MATLAB R2020a is loaded and analyzed. Three categories of characteristics were extracted: (i) the histogram of intensity, (ii) the texture, and (iii) the shape. Before the spatial relationship was considered, six first-order statistical features set the distribution values of the individual area. A total of 22 textural properties defined a grey-level co-occurrence matrix (GLCM) spatial zone pattern. The geometrical area of the tumor was created by nine elements. Moreover, the extracted imagery compromised 6 traits representing tumor intensity, 9 shape characteristics, and 29 textural features, as shown in Table 2. All the features extracted were in numerical form. These features were kept and arranged in .csv format, before being imported into Python for analysis purposes.

### 2.4. Grid Search Optimization Algorithm 

**Figure 3:**
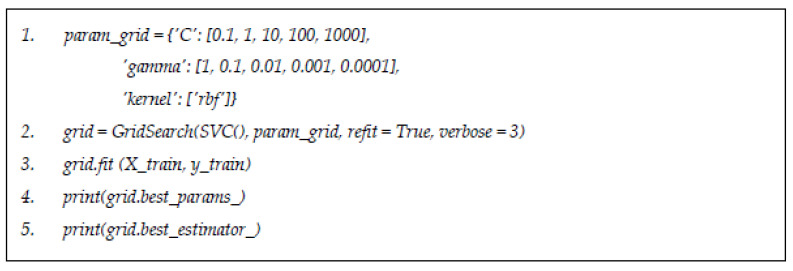
Command for parameter and estimator for support vector machine classifier.

Machine learning classifiers considered in this study include the following: naïve Bayes (NB) and support vector machine (SVM), which were trained to identify the best hyperparameters and configurations by applying best estimator methods. Python Scikit-Learn offers an effective method to carry out the grid search method in optimizing the hyperparameters on each classifier considered. This is indeed a useful tool for inexperienced data scientists to obtain recommendations for configuration parameters for selected algorithms. For example, Figure 3 shows the codes for identifying the best parameters for decision tree algorithm by using grid search estimator.

### 2.5. TPOT Model Selection

**Figure 4:**
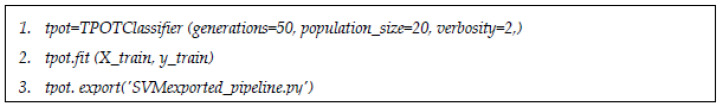
Script for TPOT classifier with only support vector machine.

TPOT was used in the classification mode in this research, with 50 generations and 20 population size sets to run. Figure 4 shows the script of algorithm for default TPOT classifier without any restriction in choosing classifiers. Both mutation and crossover were set to default. TPOT configuration can be changed according to any desired ML classifier. 

TPOT-based model selection for radiomics features was developed by using several configurations with the same classifiers considered in grid search optimization algorithm (Model 1–4): default configuration with all data operators and ML classification models (Model 1), wherein the algorithm for TPOT was implemented without any readjustment, controlled configuration with only the NB classifier including all data transformers and selectors (Model 2), controlled configuration with only SVM classifier including all data transformers and selectors (Model 3), and controlled configuration with only ANN-MLP classifier including all data transformers and selectors (Model 4). There was no restriction for TPOT iteration to choose the best pipeline and model. The integration of classifiers for MLP, SVM, and NB in this research can be found in the documentation (http://epistasislab.github.io/tpot/using/#built-in-tpot-configurations, accessed on 5 April 2020).

### 2.6. Experimental Setup

In this study, 120 TPOT experiments were performed, corresponding to 30 repetitions for the data set on each of the 4 configurations mentioned previously. Train and test split were set to 80% to 20% for training and testing data sets, respectively, with fivefold cross-validation. Across all experiments, TPOT were allowed to train to completion by terminating training after 35 generations with no improvement to the Pareto front scores, and each generation contained 50 individual pipeline trees. 

A comparison was made between TPOT-based model selection and exhaustive grid search parameter tuning of NB classifier (Model 5), SVM classifier (Model 6), and ANN-MLP classifier (Model 7). SVMs were first explained by Vladimir Vapnik, and the good performances of SVMs have been noticed in many pattern recognition problems. SVMs can indicate better classification performance when they are compared with many other classification techniques that are used for the prognosis and diagnosis of cancer. 

On the other hand, NB is a supervised ML model that uses naïve Bayes algorithm for the purpose of classification. The algorithm computes the joint distribution p(a,b) of the extracted features a and the class labels b given by p(a|b) p(b), and then learns the parameters of model [24] by maximizing its likelihood function. ANN can be expressed in terms of a biological neuron system, especially since it is similar to a human brain process system. It consists of a lot of nodes that connect each node [11]. ANN has the ability to model typical and powerful non-linear functions. It consists of a network of a large number of artificial neurons. Each of these combinations comprise input/output characteristics that perform a local mathematical function. The function could be a computation of weighted sums of inputs that generates an output if it goes beyond a given threshold value. The output could be an input to other neurons in the network. This transaction iterates until the latest output is produced.

The grid search optimization method was implied to all the ML classifiers with the best performance that were previously generated by TPOT pipelines. Various performance evaluations including accuracy, the area under the curve (AUC), precision, and recall, along with model complexity (number of transformational steps), were recorded for all ML pipelines. 

## 3. Results

### 3.1. Classification Accuracy of Model from TPOT and GS Optimization

TPOT configuration obtained a greater classification accuracy score compared to configuration from grid search hyperparameter tuning method. Markedly, default TPOT, or TPOT that was figured by GP-based AutoML system without any restriction, outperformed the other configuration with the highest accuracy score, as shown in Table 3.

**Figure 5:**
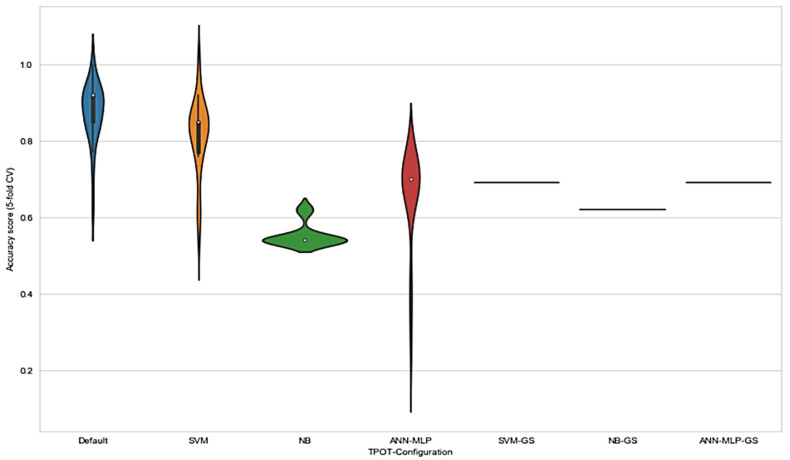
Distribution of accuracy scores for TPOT and grid search hyperparameter tuning deployed in various configurations on the radiomics data set extracted. Each distribution was of 30 experiments using the same initial TPOT configuration.

These observations are consistent with the principle of GP-based AutoML system where the evolution without any restriction acquired the best pipelines using the available set of operators and eliminated those that showed worse performances (TPOT NN). The results in Figure 5 show that the range of accuracy score varied in each configuration. Notably, the range of accuracy score for default TPOT was the highest, followed by SVM-TPOT and ANN-MLP-TPOT; however, NB-TPOT achieved the lowest range, even when compared with GS configuration. This can be explained by referring to the lack of hyperparameters in NB classifier. Therefore, the iteration of GP-AutoML system in finding the best pipeline was more constricted and challenging. The grid search method deployed in SVM-GS, NB-GS, and ANN-MLP-GS acquired a lower accuracy score than TPOT configurations. Since the accuracy scores obtained by grid search method were consistent, there were no ranges recorded for these configurations. On the basis of the results, we found that NB-GS performance was the lowest compared to SVM-GS and ANN-MLP-GS. As mentioned earlier, NB classifier comprised no hyperparameter that could be tuned to improve the result. Although it performs well with small amounts of training data, and scales well to large data sets, NB often relies on an often-faulty assumption of equally important and independent features that can somehow affect the performance of the classifier itself. 

However, SVM-GS and ANN-MLP-GS showed better performances than NB-GS, even though these two classifiers were still not good enough to outperform classifiers based on TPOT configuration.

On the basis of the observations, we found that there was a significant difference (*p* < 0.05) between the classifiers, suggesting that the accuracy between the configured classifiers can be improved by choosing the best configuration. In this study, default TPOT showed the highest results in all metrics: accuracy, precision, recall, and ROC score. This was due to the pre-processor and pipelines that were chosen by using GP process. A complete pipeline equipped with suitable pre-processor and feature selector were chosen accordingly on the basis of the input data; hence, the result can be improved with an effective pipeline. 

### 3.2. Selected Model from TPOT-Based Optimization 

Table 4 provides the model selection comparative analysis of the TPOT optimization process and the grid search parameter tuning of all models. The result of TPOT optimization for Model A1 showed a training accuracy of 0.923. The pipeline for Model A consisted of only an operator (principal component analysis (PCA)) and random forest (RF) as ML classifier.

TPOT optimization for Model A2 assembled a pipeline with two pre-processors (concatenates of two function transformers with feature union and concatenates of two stacking estimator (SEs) with the product of pre-processor before) and accuracy of 0.846. TPOT optimization for Model A3 had only classifiers without any pre-processor or tuned hyperparameter. Model A3 achieved an accuracy of 0.615. Model A4 was selected during ANN-MLP-TPOT classifier optimization and had no relevant pre-processor; however, the hyperparameter included was tuned to fit the model. Model A4 acquired an accuracy of 0.692. Grid search parameter tuning (hyperparameters tuned are shown in Table 5) for SVM, NB, and ANN-MLP reported notably lower accuracy performance compared to the accuracy achieved in the TPOT optimization model (accuracy of 0.692–0.615). The best performance for the model was proven to be Model A1, selected by the TPOT optimization with the default configuration.

**Figure 6:**
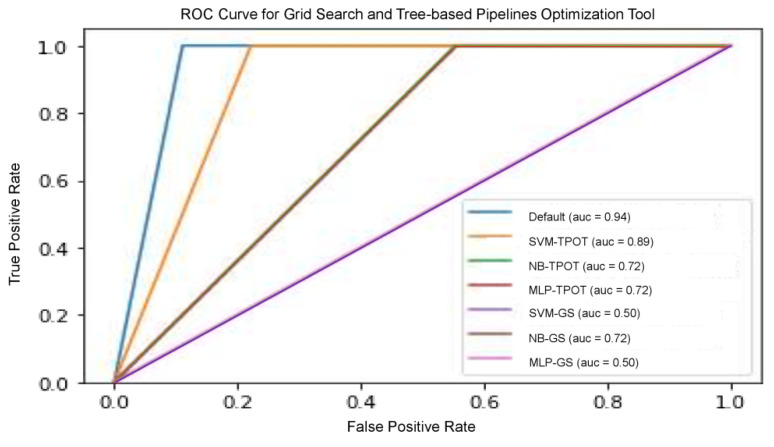
ROC curve for grid search and tree-based pipeline optimization configuration on various ML classifiers.

We examined the predictive ability of several other models to improve the validity of the results obtained from these models, including precision, recall, and threshold-based measurements. Precision is one of the primary metrics that describe the ability of the model to assess samples that are not positive. Recall (sensitivity) is often accompanied by accuracy—this helps to determine all positive samples. These metrics are categorized as a single threshold, which means that they cannot specify a set of judgement parameters because they are specified for a single decision threshold. Nevertheless, this problem could be remedied with the plotting of different ROC curves. It is commonly used because the classifier threshold varies. The true positive rate (number of correctly classified samples) is shown to differ with the wrong positive rate (number of poorly classified samples), as shown in Figure 6. The RF model optimized by TPOT posted the highest results, including accuracy, precision, recall, and ROC score compared to other models, since RF is well established in the radiomics community for performing well. NB-TPOT (green-coloured line), MLP-TPOT (red-coloured line) and NB-GS (brown-coloured line) acquired the same value for ROC-AUC, therefore, all the curve overlapped each other resulting in only one line visible. The same applied to SVM-GS (purple-coloured line) and MLP-GS (pink-coloured line), where same ROC-AUC resulting in overlapped curve.

### 3.3. Pipeline Complexity on the Performance of Model Selection 

Further investigation on the effect of pipeline complexity on the performance of the model selection was conducted. We hypothesized that a more complex model is more likely to generate better performance compared to a less complex model (Table 6). Pipeline complexity is referred to as the number of pre-processors and operators included in building a pipeline. The greater the number of pre-processors and operators used in a pipeline, the higher the pipeline complexity. We evaluated the stability of the models with sensitivity analysis, wherein we excluded each pre-processing operator continuously with pipeline reduction (Pr) to analyze the performance of all the selected models. The accuracy and ROC AUC performance declined for each classifier after the pre-processors were eliminated consecutively—this was clearly shown in the table. As the pipeline reduced from Pr-1 to Pr-3, the result declined from the original pipeline given by TPOT iteration. This proves that the data set may generate intricate non-linear relationships among features, and therefore complexity combination of data transformers is necessary to explore these relationships. Hence, we observed a clear decrease in the complexity of all output metrics, reflecting the general trend for increased complexity.

By referring to the outcome of the selected models, we deduced that an appropriate choice and optimization of each pipeline are extremely important in achieving maximum performance score of the models. To make an unbiased comparison between TPOT optimization and grid search-based model selection approach, we decided to assess the performance of all models in various combinations of SS and RFE pre-processors, as shown in Table 7. With the addition of SS operator and RFE selector consecutively in all models, there was a slight increment of accuracy in the performance for all selected models. However, there was no significant change when SS operator was added into the pipeline.

There are several models that were excluded from RFE, including ANN-MLP and NB classifier, since they do not provide any logic that could enable us to implement RFE on it. NB works by determining the conditional and unconditional probabilities associated with the features and predicts the class with the highest probability. Thus, there are no coefficients computed or associated with the features used to train the model. MLP, on the other hand, is a form of neural network architecture and involves detail adjustment on the architecture itself. Hence, random permutation was adapted as another pre-processor to observe whether the results improved with the shuffling of the features randomly. However, the results showed no improvement, which suggests that extra measures in adjusting the architecture are needed. On the other hand, the result for SVM increased drastically when SS and RFE were added. 

**Figure 7:**
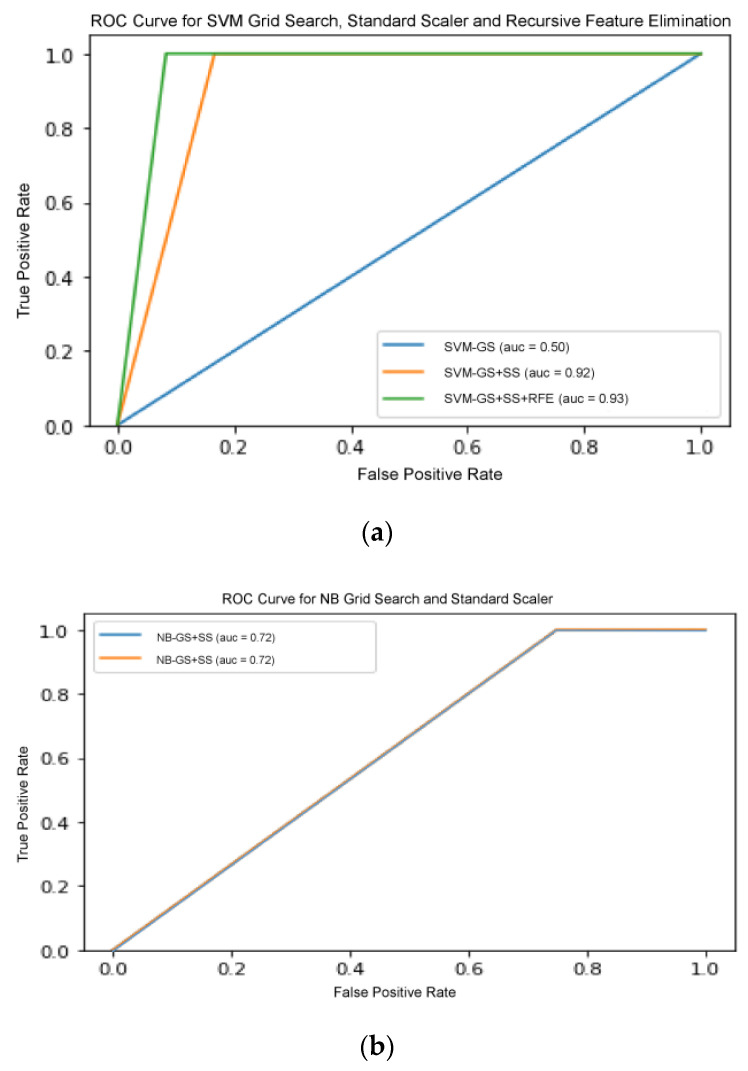

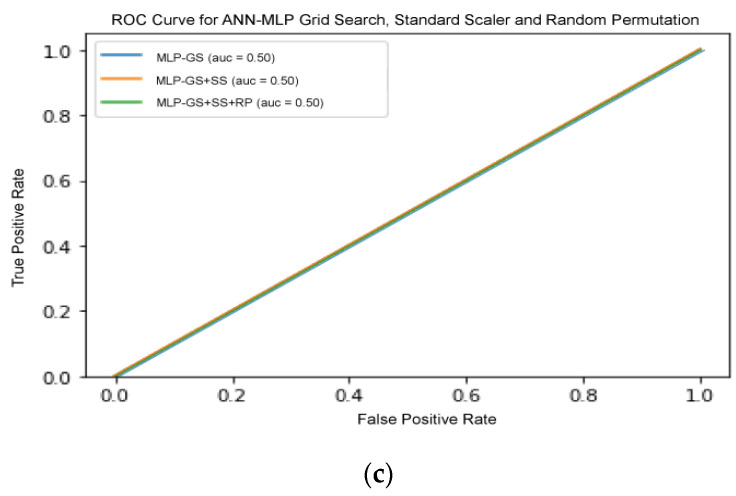
(**a**) ROC curve for SVM with GS, standard scaler, and recursive feature elimination. (**b**) ROC curve for NB with GS and standard scaler. (**c**) ROC curve for SVM with GS, standard scaler, and random permutation.

This shows that the increase of pipeline complexity can help in improving the performance of a model. Figure 7 shows the ROC curve for all three classifiers with increasing pipeline complexity. Figure 7b,c shows that the curves in each figure acquired the same ROC-AUC, consequently the curves overlapped each other. Therefore, only one line are visible in both figure. This comparative analysis deduced that appropriate selection and optimization of the data pre-processing operators were important in improving the accuracy of performance. Although the inclusion of certain different pre-processors enhanced GS optimization compared to the ML algorithm tuning itself, the best total ML solution was still offered by TPOT agnostic optimization.

### 3.4. Time Efficiency of TPOT

**Figure 8:**
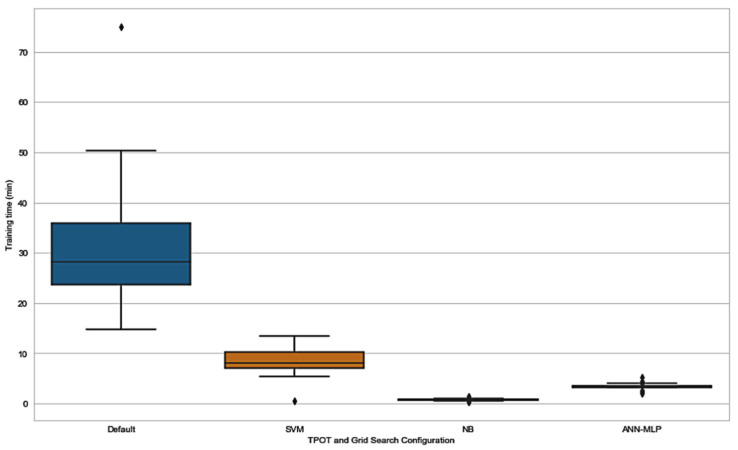
CPU clock time distributions for training TPOT on each configuration.

In addition to exploring the effect of application of TPOT as a hyperparameter and pipeline optimizer, we explored the time consumed by all the TPOT configurations. As expected, default TPOT consumed more time in order to be trained compared to TPOT restricted to only one classifier. The results in Figure 8 and Table 8 show that time taken for training in SVM-TPOT, NB-TPOT, and ANN-MLP-TPOT was statistically different when compared to the time taken by default TPOT. This suggests that there is a huge difference in training time between all TPOT configurations and default TPOT, as default TPOT consumes a large amount of time. Configuration from the GS method was not taken into account because the time taken is too short and irrelevant to be considered in this research.

## 4. Discussion

### 4.1. Excellent Compatibility between TPOT Configuration and Radiomics Features

Recently, TPOT has been extensively tested on many specialized classification tasks, especially in medical diagnosis, which comprises an important topic that we have prioritized for future exploration. On the basis of minimum assumptions concerning the model selection used by TPOT, we found that the agnostic approach presented better clinical predictive potential, especially if the mechanistic relationship among various characteristics was unknown. In this research, we used two TPOT optimization solutions together, with a complete list of pre-processors and classifiers. Our configurations were reduced, with a complete pre-processor list and a preferred classifier (SVM, NB, and NN-MLP). We discovered that default TPOT developed model pipelines that can overcome any optimization strategy, including the reduced TPOT configuration and grid search with or without pre-processors. This shows that the choice of the ML algorithm and their parameters (for example, Table 3 shows that the elimination of the RF classification by the pre-processor slightly decreased the accuracy of 8.4 percent (*p* > 0.05)). This was also shown by optimization of the grid search (Table 4). Alternatively, extensive computer resources are needed for the selection of the agnostic model via the grid search approach. Some pre-processors will massively increase the runtime. Therefore, an agnostic approach toward grid search models cannot be feasible with modern computational resources and stochastic methods of search, such as genetic programming, potentially the most suitable approach, especially when dealing with radiomics features.

### 4.2. Evaluating the Trade-Off between Model Performance and Training Efficiency

The amount of time needed to train a pipeline is an important pragmatic consideration in the real-world applications of ML. This certainly extends to the case of AutoML. The parameters we used for TPOT include somewhere between 35 and 50 training generations, with a population size of 50 in each generation; therefore, we evaluated several thousand candidate pipelines—each of which consisted of a variable number of independently optimizable operators—for every experiment (of which there were 120 in the present study). As shown in Table 5, we generally expected a default TPOT configuration to train for almost an hour, or even slightly over an hour, depending on the generation and population size. Computational time will increase as the generation or the population size increases. In most cases, the performance of a model improved with an increase in the population size and generation. Users will have to determine, on an individual basis and dependent on the use case, whether the potential accuracy increase of, at most, several percentage points is worth the additional time and computational investment inherent to the GP-AutoML process.

Nevertheless, the results acquired from this research show that it is unlikely for the TPOT pipeline to perform worse than the non-TPOT pipeline or conventional hyperparameter tuning. In ‘mission critical’ settings where training time is not a major concern, TPOT can be expected to perform and help researchers to yield better performance in finding an effective ML pipeline.

### 4.3. Limitations and Improvement for Future Works

The main limitations of using TPOT in this research were the computational limit and cost incurred by this method. GP radiomics optimization methods are typically criticized for optimizing a large population of solutions, which can sometimes be slow and wasteful for certain optimization problems [24]. However, it is possible to turn GP’s purported weakness into a strength by creating an ensemble out of the GP populations. Since the result of the controlled TPOT configuration-based model achieved showed no improvement when compared to GS based model, it is recommended that researchers should experiment with a wide range of hyperparameters for each GS-based model. In this research, the efficiency of agnostic model selection using AutoML TPOT for cancer prediction was demonstrated by using data set radiomics. 

## 5. Conclusions

In this study, we showed that the default TPOT model for the selected data set produced classification pipelines that exceeded the performance of the controlled TPOT configuration-based model and grid search optimization-based model. The RF classifier showed an outstanding outcome amongst the models in combination with just two pre-processors, with a precision of 0.83. The grid search optimized for SVM classifiers generated a difference of 12% in comparison, while the other two classifiers, NB and NN-MLP, generated a difference of almost 39%. This study demonstrated the efficiency of agnostic model selection using AutoML TPOT for cancer prediction by using data set radiomics.

## Figures and Tables

**Table 1:** Image details from The Cancer Imaging Archive (TCIA) data set.

TCIA—Digital Database for Screening Mammography (DDSM)	
**Breast Density**	(1): 50
	(2): 172
	(3): 112
	(4): 44
**Image View**	CC: 177
	MLO: 201
**Mass Shape**	Architectural distortion: 23
	Asymmetric breast tissue: 5
	Focal asymmetric density: 6
	Irregular: 113
	Irregular architectural distortion: 7
	Irregular asymmetric breast tissue: 1
	Lobulated: 79
	Lobulated Irregular: 1
	Lobulated Lymph Node: 1
	Lymph Node: 9
	Oval: 91
	Oval-lobulated: 1
	Round: 41
**Mass Margins**	Circumscribed: 87
	Circumscribed ill-defined: 2
	Circumscribed microlobulated: 1
	Circumscribed microlobulated ill-defined: 3
	Circumscribed-obscured: 3
	Circumscribed-obscured ill-defined: 4
	Circumscribed-spiculated: 1
	Ill-defined: 92
	Ill-defined-spiculated: 5
	Microlobulated: 21
	Microlobulated ill-defined: 2
	Obscured: 50
	Obscured-Circumscribed: 2
	Obscured Ill-defined: 5
	Obscured Ill-defined-spiculated: 1
	Spiculated: 82

**Table 2:** Features extracted using grey-level co-occurrence matrix (GLCM), first order statistics, and shape features.

GLCM Features	First Order Statistics	Shape Features
GLCM_Autocorrelation	Mean	Area
GLCM_Contrast	Variance	Major Axis Length
GLCM_Correlation	Skewness	Minor Axis Length
GLCM_Correlation	Kurtosis	Eccentricity
GLCM_Cluster Prominence	Energy	Orientation
GLCM_Cluster Shade	Entropy	Convex Area
GLCM_Dissimilarity		Equivdiameter
GLCM_Energy		Solidity
GLCM_Entropy		Perimeter
GLCM_Homogeneity		
GLCM_Homogeneity		
GLCM_Maximum probability		
GLCM_Sum of squares		
GLCM_Sum average		
GLCM_Sum variance		
GLCM_Sum entropy		
GLCM_Difference variance		
GLCM_Difference entropy		
GLCM_ Information measure of correlation1		
GLCM_ Information measure of correlation2		
GLCM_Inverse difference normalized		
GLCM_ Inverse difference moment normalized		

**Table 3:** Accuracy score for various TPOT and grid search method configuration.

Accuracy for TPOT Configuration							Two-Tailed *p*-Value	
Default TPOT	SVM-TPOT	NB-TPOT	MLP-ANN-TPOT	SVM-GS	NB-GS	ANN-MLP-GS	Wilcoxon	Levene
0.923	0.846	0.615	0.692	0.692	0.615	0.692	<0.05	<0.05

**Table 4:** Comparative analysis of the TPOT optimization of the selected model with various metrics.

Model	Accuracy	Precision	Recall	ROC AUC	Pipeline Complexity	Two-Tailed *p*-Value (Compare with Accuracy of Default TPOT Configuration) Using Wilcoxon Rank Test
A1. Default TPOT (RF)	0.923	0.83	1.00	0.937	2	-
A2. SVM-TPOT	0.846	0.71	1.00	0.889	4	>0.05
A3. NB-TPOT	0.615	0.44	1.00	0.722	1	<0.05
A4. NN-MLP-TPOT	0.692	0.44	1.00	0.722	1	<0.05
A5. SVM-GS	0.692	0.44	1.00	0.500	1	<0.05
A6. NB-GS	0.615	0.44	1.00	0.722	1	<0.05
A7. ANN-MLP-GS	0.692	0.44	1.00	0.500	1	<0.05

**Table 5:** Parameters and pre-processor operators of each configuration.

Model	Parameters and Pre-Processor Operators Chosen
A1. Default TPOT (RF)	PCA (iterated_power = 10, svd_solver = “randomized”) and RandomForestClassifier (bootstrap = False, criterion = “entropy”, max_features = 0.1, min_samples_leaf = 2, min_samples_split = 6, n_estimators = 100)
A2. SVM-TPOT	MakeUnion (FunctionTransformer(copy) + FunctionTransformer(copy)) StackingEstimator (estimator = LinearSVC (C = 0.1, dual = False, loss = “squared_hinge”, penalty = “l1”, tol = 0.0001)) StackingEstimator(estimator = LinearSVC(C = 0.5, dual = False, loss = “squared_hinge”, penalty = “l2”, tol = 0.01)) LinearSVC(C = 5.0, dual = False, loss = “squared_hinge”, penalty = “l1”, tol = 0.001)
A3. NB-TPOT	GaussianNB()
A4. ANN-MLP-TPOT	MLPClassifier(alpha = 0.0001, learning_rate_init = 0.5)
A5. SVM-GS	Range of hyperparameter included: ‘C’: [0.1,1, 10, 100] ‘kernel’: [‘rbf’, ‘poly’, ‘sigmoid’]} ‘gamma’: [1,0.1,0.01,0.001] Selected hyperparameter: ‘C’: 0.1, ‘gamma’: 1, ‘kernel’: ‘rbf’
A6. NB-GS	-
A7. ANN-MLP -GS	Range of hyperparameter included: activation = [‘logistic’, ‘tanh’, ‘relu’] alpha = [0.0001, 0.05] hidden_layer_sizes = [(10,10,10), (20,20,20), (50,50,50)] max_iter = [100, 200] Selected hyperparameter: activation = ‘tanh’, alpha = 0.05, hidden_layer_sizes = (50, 50, 50), max_iter = 100

**Table 6:** The complexity–performance relationship for models selected by the TPOT optimization for each selected model.

Model	Accuracy	Precision	Recall	ROC AUC	Pipeline Complexity
A1. Default TPOT	0.923	0.83	1.00	0.937	2
Pr-1 (RF)	0.846	0.50	0.50	0.704	1
A2. SVM-TPOT	0.846	0.71	1.00	0.889	4
Pr-1	0.846	0.67	1.00	0.875	3
Pr-2	0.846	0.67	1.00	0.875	2
Pr-3	0.846	0.67	1.00	0.875	1
Pr-4	0.846	0.67	1.00	0.875	1
A3. NB-TPOT	0.615	0.44	1.00	0.722	1
A4. ANN-MLP-TPOT	0.615	0.44	1.00	0.722	1

**Table 7:** Comparative analysis of the grid search optimization of selected ML algorithms with SS and RFE pre-processing operators for each model.

Models	Accuracy	Precision	Recall	ROC AUC	Pipeline Complexity
SVM	0.692	0.33	1.00	0.500	1
SVM + SS	0.846	0.44	1.00	0.916	2
SVM + SS + RFE	0.846	0.44	1.00	0.927	3
NB	0.615	0.44	1.00	0.722	1
NB + SS	0.615	0.44	1.00	0.722	2
NB + SS + RFE	-				
ANN-MLP	0.692	0.44	1.00	0.500	1
ANN-MLP + SS	0.692	0.44	1.00	0.500	2
ANN-MLP + SS + RP	0.692	0.44	1.00	0.500	3

**Table 8:** Training time for all TPOT configurations.

Model	Training Time 1 (m)	Training Time 2 (m)	Training Time 3 (m)	Training Time 4 (m)	Training Time 5 (m)	Two-Tailed *p*-Value (Compare with Training Time of Default TPOT Configuration) Using Wilcoxon Rank Test
A1. Default TPOT	44.30	43.80	35.30	42.50	50.00	-
A2. SVM-TPOT	10.13	10.36	10.57	10.40	13.49	<0.05
A3. NB-TPOT	0.74	0.72	0.87	0.71	0.75	<0.05
A4. ANN-MLP-TPOT	4.00	4.11	4.35	4.31	3.28	<0.05

## Data Availability

All relevant data are included in the study.
